# An Overview of the CNS-Pharmacodynamic Profiles of Nonselective and Selective GABA Agonists

**DOI:** 10.1155/2012/134523

**Published:** 2012-01-29

**Authors:** Xia Chen, Sanne de Haas, Marieke de Kam, Joop van Gerven

**Affiliations:** ^1^Phase I Unit of Clinical Pharmacological Research Center, Peking Union Medical College Hospital, 100032 Beijing, China; ^2^Centre for Human Drug Research, 2333 CL Leiden, The Netherlands

## Abstract

Various *α*_2,3_ subtype selective partial GABA-A agonists are in development to treat anxiety disorders. These compounds are expected to be anxiolytic with fewer undesirable side effects, compared to nonselective GABA-A agonists like benzodiazepines. Several *α*_2,3_ subtype selective and nonselective GABA-A agonists have been examined in healthy volunteers, using a battery addressing different brain domains. Data from five placebo-controlled double-blind studies were pooled. Lorazepam 2 mg was the comparator in three studies. Three *α*_2,3_-selective GABAA agonists (i.e., TPA023, TPACMP2, SL65.1498), one *α*_1_-selective GABAA agonists (zolpidem), and another full agonist (alprazolam) were examined. Pharmacological selectivity was assessed by determination of regression lines for the change from baseline of saccadic-peak-velocity- (ΔSPV-) relative effect, relative to changes in different pharmacodynamic endpoints (ΔPD). SPV was chosen for its sensitivity to the anxiolysis of benzodiazepines. Slopes of the ΔSPV-ΔPD relations were consistently lower with the *α*_2,3_ selective GABA-A agonists than with lorazepam, indicating that their PD effects are less than their SPV-effects. The ΔSPV-ΔPD relations of lorazepam were comparable to alprazolam. Zolpidem showed relatively higher impairments in ΔPD relative to ΔSPV, but did not significantly differ from lorazepam. These PD results support the pharmacological selectivity of the *α*_2,3_-selective GABA-A agonists, implying an improved therapeutic window.

## 1. Introduction

Anxiety is a psychological and physiological state with somatic, emotional, cognitive, and behavioral components [1], which dominates thinking and leads to disturbance of daily functioning. Serotonergic antidepressants, either selective serotonin reuptake inhibitors (SSRIs) or serotonin-norepinephrine reuptake inhibitors (SNRIs), are currently prescribed as the 1st-line treatment for several anxiety disorders. However, the slow onset of therapeutic effect and the presence of sexual side effects prevent these drugs from more extensive use and lead to lack of treatment compliance [2]. Moreover, SSRIs/SNRIs cause transient increase of anxiety during the first few weeks of administration. All these clinical experiences provide space for the use of benzodiazepines (BZDs) in acute anxiety episodes.

Benzodiazepines are the most commonly prescribed anxiolytic drugs, although treatment guidelines generally limit their use to several weeks to prevent the occurrence of tolerance and dependence. Benzodiazepines are allosteric modulators of the GABA_A_ receptors that affect the central nervous system (CNS) as full GABAergic agonists [3]. As a consequence, these drugs have detrimental effects on alertness, memory, postural stability, and muscle tone. In loss-of-function studies conducted in point-mutated mice [4], different subtypes of GABA_A_ receptors have been found responsible for the specific aspects of benzodiazepine pharmacology: (1) *α*_1_-containing receptors are associated with sedative effects of benzodiazepines [5, 6]; (2) *α*_2_/*α*_3_-containing receptors are related to anxiolysis and analgesia [7, 8]; (3) *α*_5_-receptors are associated with cognition [9, 10]. BZDs exert their CNS actions in a concentration-related manner [11]. The anxiolytic, hypnotic, muscle relaxant, and amnesic effects of BZDs generally appear concomitantly, and the onset and duration of action of the compounds correlate closely with their pharmacokinetic properties. The effect profile of BZDs has been attributed to their non-selective agonism at the *α*_1_, *α*_2_, *α*_3_, and *α*_5_ subunit-containing GABA_A_ receptors. To improve the pharmacological and functional selectivity, novel GABAergic anxioselective compounds are evaluated using recombinant human GABA_A_ receptors during preclinical development. The GABAergic effect profile of a compound is characterized by the affinity of the ligand for the receptor and by the* in vitro *efficacy of the compound at each GABA_A_ receptor subtype. In the past years, several partial GABA_A_ agonists have been developed, which have a relatively high *in vitro *efficacy at *α*_2_/*α*_3_ subtypes compared with *α*_1_ or *α*_5_ subtypes. Such *α*_2_/*α*_3_ subtype-selective partial GABA agonists are anticipated to have favorable therapeutic effect and to be less sedating or cognition impairing (Table 1).

Based on nonclinical investigations with* in vitro* assays and animal models of anxiety, the human pharmacology of novel GABAergic agents is approached through sequential clinical studies regarding pharmacokinetics, receptor occupancy, and pharmacodynamics (PD) in healthy volunteers. Direct links have been proposed between plasma drug concentration and receptor occupancy [4], as well as between plasma drug concentration and pharmacodynamic parameters [12–15]. Such pharmacokinetic/pharmacodynamic (PK/PD) relationships warrant the assessment of surrogate biomarkers in healthy volunteers treated with single doses of selective novel GABAergic compound(s).

More than 170 pharmacodynamic tests or test variants have been developed to assess the CNS effects of benzodiazepines [11]. De Visser et al. analyzed the interstudy consistence, sensitivity, and pharmacological specificity of the frequently used biomarkers. Saccadic peak velocity (SPV) and visual analogue scale of alertness (VAS_alertness_) were identified as the most sensitive parameters for benzodiazepines. Both tests showed consistent effects to a variety of benzodiazepines at different doses.

During the past fifteen years, the Centre for Human Drug Research (CHDR) has established a selection of computerized neuropsycho-pharmacodynamic tests called the Neurocart battery. The components of this battery target a variety of neurophysiological and/or neuropsychological domains (Table 2). Of this battery, adaptive tracking, saccadic eye movements, and body sway were proved sensitive to the sedating effects of sleep deprivation [16], as well as benzodiazepines and other GABAergic drugs. In the recent years, the Neurocart battery was used in a series of phase I studies to assess CNS pharmacodynamics of partial *α*_2,3_ subtype selective GABA_A_ agonists. Both nonselective and/or selective GABA_A_ agonists were administered as single oral dose to healthy volunteers. Clear distinctions of effect profile were observed in these trials [12–14]. The objective of this paper was to characterize the pharmacodynamic effect profiles of novel anxioselective GABA_A_ agonists and identify suitable biomarkers to distinguish *α*_2,3_ subtype-specific GABA_A_ agonists from full GABA_A_ agonists like benzodiazepines.

## 2. Methods

Five clinical studies, all of which are published [12–15, 17], were conducted at the CHDR in healthy volunteers after approval from the Ethics Review Board of Leiden University Medical Centre. All subjects provided written inform consent for study participation. Each trial was designed as single-dose, cross-over or parallel-armed, randomized, double-blind, placebo- and/or positive-controlled study. The subjects took single oral doses of a selective GABAergic compound, placebo-, and/or a nonselective benzodiazepine. Three studies used lorazepam 2 mg as a positive control, whereas in the studies with zolpidem 10 mg and alprazolam 1 mg, these drugs were the only GABAergic study medications. Data of all studies came from the same research center and were pooled from the studies-specific electronic databases kept by the center.* In vitro* pharmacological parameters of novel compounds were extracted from the Investigator's Brochures and published articles. These parameters provide reliable information about the subtype selectivity of each compound, but it is more difficult to compare the pharmacological properties between the drugs. Due to the diversity of cell types and GABA_A_ receptor homologies used in the whole-cell patch clamping assays, the links between* in vitro* pharmacology and human* in vivo* effects are considered less quantitative and semiquantitative comparisons are preferred.

### 2.1. Treatments

Three novel drugs designed to be *α*_2,3_ subtype selective were dosed in three of the above-mentioned studies (for each dose group, the number of study participants is provided in parentheses): TPA023 0.5 mg, 1.5 mg (*n* = 12) [12]; TPACPM2 (MK0343) 0.25 mg, 0.75 mg (*n* = 12) [13]; SL65.1498 2.5 mg, 7.5 mg, and 25 mg (*n* = 20) [14]. Zolpidem is a hypnotic with a high affinity for *α*_1_-subtypes, and alprazolam is a nonselective GABAergic anxiolytic. Zolpidem 10 mg (*N* = 14) [15] and alprazolam 1 mg (*N* = 20) were administered in another two studies, respectively.

### 2.2. Pharmacodynamic Assessments

#### 2.2.1. Saccadic Eye Movement

Saccadic eye movements are very sensitive to a variety of mostly CNS-depressant drugs [18, 19]. Saccadic peak velocity has been shown to be closely related to the anxiolytic properties of benzodiazepines [4]. Since partial *α*_2,3_-subtype-selective GABA_A_ agonists are developed to be anxiolytic, it was expected that these compounds would reduce saccadic peak velocity, similar to what is typically observed with benzodiazepines. Therefore, saccadic peak velocity was used as a biomarker for the anxiolytic properties of the GABA_A_ agonists, to which all other pharmacodynamics effects were compared in this meta-analysis. Recording and analysis of saccadic eye movements was conducted with a microcomputer-based system for sampling and analysis of eye movements. The program for signal collection and the AD converter were from Cambridge Electronic Design (CED Ltd., Cambridge, UK), the amplifiers were supplied by either Nihon Kohden (Nihon Kohden, Life Scope EC, Tokyo, Japan) or Grass (Grass-Telefactor, An Astro-Med, Inc. Product Group, Braintree, USA), and the sampling and analysis scripts were developed at CHDR (Leiden, The Netherlands).

#### 2.2.2. Smooth Pursuit

The same systems as used for saccadic eye movements were also used for measuring smooth pursuit. For smooth pursuit eye movements, the target moves sinusoidally at frequencies ranging from 0.3 to 1.1 Hz, in steps of 0.1 Hz. The amplitude of target displacement corresponds to 22.5 degrees eyeball rotation to both sides. Four cycles were recorded for each stimulus frequency. The method has been validated at CHDR by Van Steveninck based on the work of Bittencourt et al. [20] and the original description of Baloh et al. [21].

#### 2.2.3. Visual Analogue Scales (VASs)

Visual analogue scales as originally described by Norris [22] were used previously to quantify subjective effects of benzodiazepines [19]. From the set of sixteen scales, three composite factors were derived as described by Bond and Lader [23], corresponding to alertness, mood, and calmness. These factors were used to quantify subjective drug effects.

#### 2.2.4. Body Sway

The body sway meter measures body movements in a single plane, providing a measure of postural stability. Body sway was measured with an apparatus similar to the Wright ataxiameter, which integrates the amplitude of unidirectional body movement transferred through a string attached to the subject's waist. Two-minute measurements were made in the anteroposterior direction with eyes open and closed, with the subject standing comfortably on a firm surface with their feet slightly apart. The method has been used before to demonstrate postural instability due to benzodiazepines [24, 25].

#### 2.2.5. Adaptive Tracking

The adaptive tracking test as developed by Hobbs and Strutt was used, according to specifications of Atack et al. [26]. The adaptive tracking test is a pursuit-tracking task. A circle of known dimensions moves randomly across a screen. The test subject must try to keep a dot inside the moving circle by operating a joystick. If this effort was successful, the speed of the moving circle increases. Conversely, the velocity was reduced if the test subject cannot maintain the dot inside the circle. The adaptive tracking test is a measure of visuomotor coordination that has proved to be very sensitive of various psychoactive drugs [27].

Table 3 summarizes the pharmacodynamic tests used in the different studies.

### 2.3. Statistical Analysis

Individual graphs are generated for each pharmacodynamic variable (*y*-axis) versus SPV change from baseline (*x*-axis). Summary graphs are generated with lorazepam and one other treatment per graph, for all GABAergic treatments.

A regression analysis of change from baseline of body sway (ΔSway), tracking (ΔTrack), VAS alertness (ΔVAS_alertness_), or VAS calmness (ΔVAS_calmness_) against the change from baseline of SPV (ΔSPV) was performed with a mixed effect model on the available individual data. The fixed factor was the GABAergic treatment and treatment by saccadic peak velocity, while the random factors were subject slope and intercept. The values of body sway were analyzed after log-transformation, while the other parameters were taken without transformation. The estimates of the slopes of the linear relations of these ΔSPV-relative effect profiles were compared between each dose of subtype-selective GABA_A_ agonists and lorazepam. The estimates of slopes, their estimated difference, and the *P* values were tabulated. Thereafter, summary plots were generated, combined with the population regression line as calculated in the regression.

All statistical analyses were carried out with SAS for Windows v9.1.3 (SAS institute, inc., Cary, NC, USA).

## 3. Results

### 3.1. ΔSPV-ΔSway Relation (Δ = Change from Baseline)

**Figure 1:**
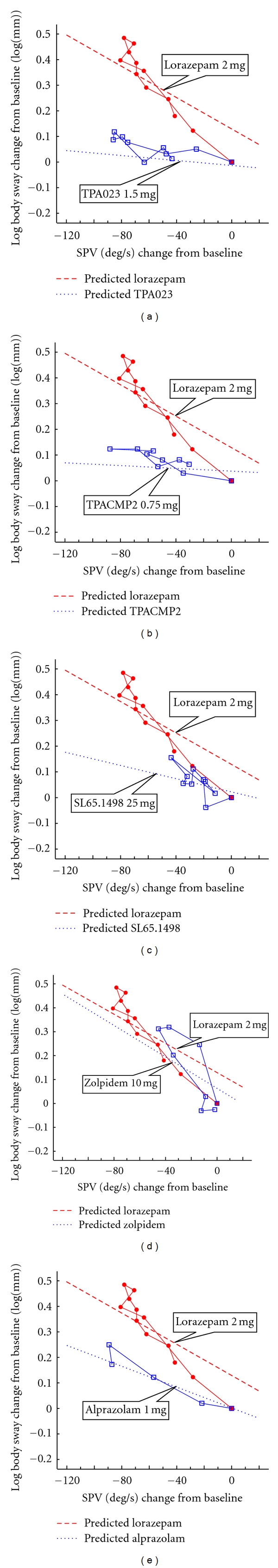
ΔLogSway (log mm)-ΔSPV (deg/sec) relative effect profile of TPA023 1.5 mg, TPACMP2 0.75 mg, SL65.1498 25 mg, zolpidem 10 mg, and alprazolam 1 mg versus lorazepam 2 mg, respectively. (Blue open square: investigational compound; red closed circle: lorazepam 2 mg; blue dot line: the comparator drug; red dash line: lorazepam 2 mg.)

Average changes from baseline of body sway against SPV within the investigational time course (i.e., 6 hours after dose) were plotted by study.  Figure 1 demonstrates clear distinctions between the ΔSPV-relative effect profile of lorazepam 2 mg and most doses of the *α*_2,3_-subtype selective compounds (i.e., TPA023 1.5 mg, TPACMP2 0.75 mg). The full GABA_A_ agonist alprazolam is similar to lorazepam. The slope of the ΔSPV-ΔSway plots for zolpidem is slightly steeper than for lorazepam.

As was revealed by the statistical analysis using the mixed linear model (Table 4), the estimated differences of the slope of regression lines are statistically significant between lorazepam and the *α*_2,3_ subtype selective partial GABAergic treatment of TPA023 1.5 mg, TPACMP2 0.75 mg, and SL65.1498 25 mg. There is no statistically significant difference between the slopes for lorazepam and alprazolam, and the difference with zolpidem suggested by the average plots (Figure 1) is not confirmed by the model (Table 4).

### 3.2. ΔSPV-ΔVAS_alertness_ Relation

**Figure 2:**
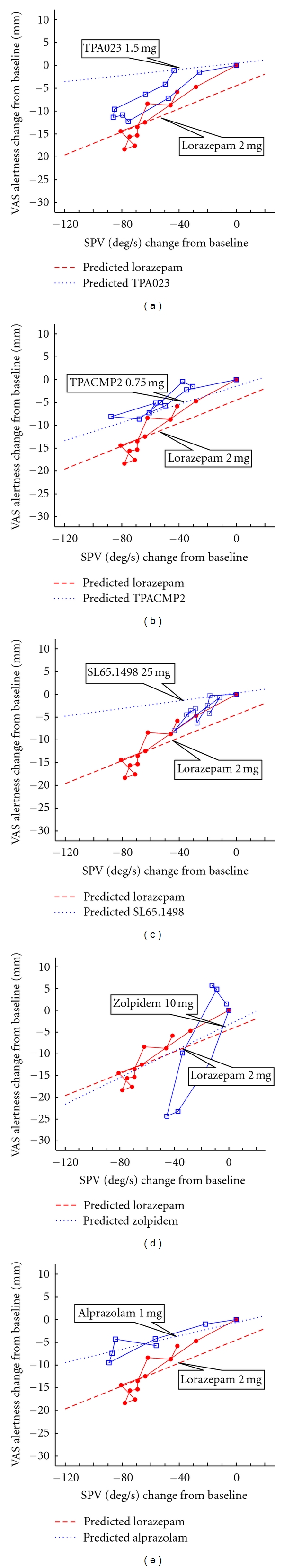
ΔVAS_alertness_-ΔSPV relative effect profile of TPA023 1.5 mg, TPACMP2 0.75 mg, SL65.1498 25 mg, zolpidem 10 mg, and alprazolam 1 mg versus lorazepam 2 mg, respectively. (Blue open square: investigational compound; red closed circle: lorazepam 2 mg; blue dot line: the comparator drug, red dash line; lorazepam 2 mg.)

Figure 2 plots the average values of ΔVAS_alertness_ versus ΔSPV obtained from individual subjects per study. As was found for the ΔSPV-ΔSway relations, a similar difference to lorazepam was observed with novel subtype selective GABAergic compounds. The slopes of the regression line of the ΔSPV-ΔSway relation for TPA023 1.5 mg and SL65.1498 25 mg are statistically shallower than the slope for lorazepam, respectively. No statistical differences can be demonstrated for TPACMP2 0.75 mg, alprazolam 1 mg, or zolpidem 10 mg.

### 3.3. ΔSPV-ΔSmooth Relation

**Figure 3:**
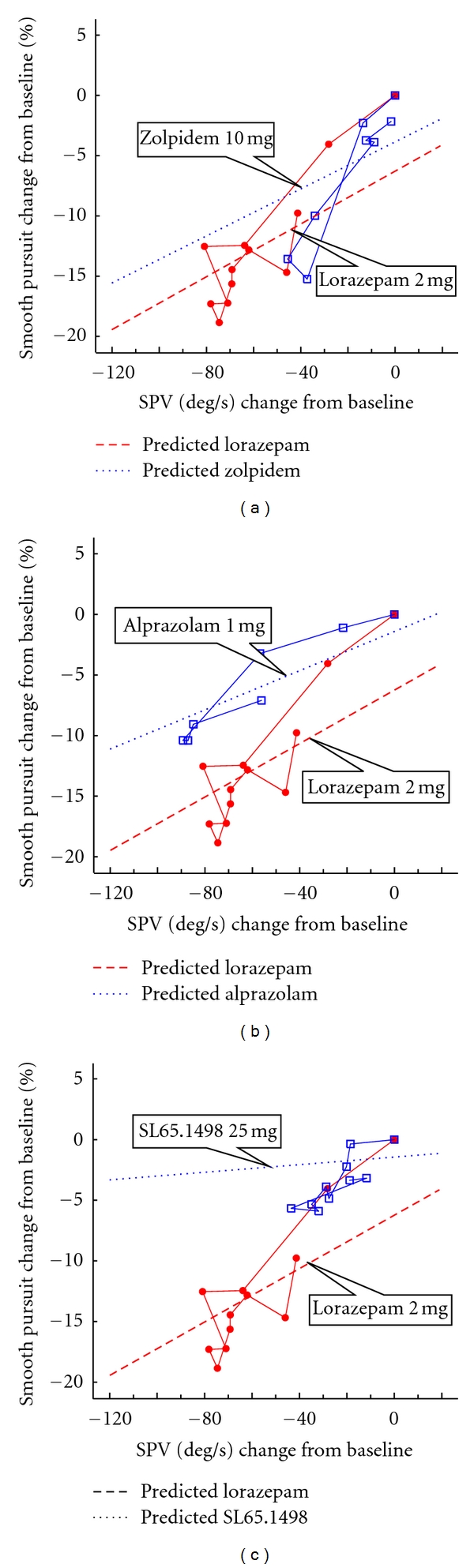
ΔSmooth-ΔSPV relative effect profile of SL65.1498 25 mg, zolpidem 10 mg, and alprazolam 1 mg versus lorazepam 2 mg, respectively. (Blue open square: investigational compound; red closed circle: lorazepam 2 mg; blue dot line: the comparator drug, red dash line; lorazepam 2 mg.)

Figure 3 and Table 4 provide the ΔSPV-relative effect profiles and the slopes and intercept for smooth pursuit after alprazolam, zolpidem, and SL65.1498. Smooth pursuit was not determined with the other partial agonists. Statistically significant differences are found in the slope of regression lines with SL65.2498 25 mg. Zolpidem and alprazolam show comparable slopes to lorazepam.

### 3.4. ΔSPV-ΔPD Relations versus In Vitro Pharmacological Properties

This analysis surmises that comparisons of ΔSPV-ΔPD profiles represent the underlying pharmacological characteristics of subtype selective and nonselective GABA_A_ agonists. A further corroboration of this approach could be provided by a comparison of ΔSPV-ΔPD profiles with the underlying pharmacological properties. This should be possible in principle, but the quantitative preclinical information provided in Table 1 was derived from different sources which in themselves were incomparable, despite the fact that all programs used oocyte-clamp assays to characterize the different GABAergic compounds. Some of these differences could be diminished by calculation of the ratio of relative efficacy on the *α*_1_  GABA_A_ subunit to that on the *α*_2_ subunit, as a benchmark of *α*_2_-specificity of the GABAergic compounds. This calculated ratio is provided in Table 1. Although the number of compounds in this overview is too small for any meaningful statistical evaluation, it is interesting that the four compounds for which this could be calculated showed a close relationship between *α*_1_/*α*_2_-efficacy ratios and ΔSPV-ΔVAS alertness ratios with borderline statistical significance (*r* ^2^ = 0.86, two-sided *P* = 0.0727). Due to the absence of *in vitro* pharmacological data and the difference of experimental settings of the trail with alprazolam, alprazolam was not included into the present analysis.

## 4. Discussion

This analysis was performed to explore the central nervous system (CNS) effects of various GABAergic agents and characterize the pharmacodynamic effect profiles of these compounds in healthy volunteers and correlate such profiles to their pharmacological properties.

A battery of CNS pharmacodynamic tests was administered to healthy volunteers who were dosed with GABAergic compound(s). The composition of the CNS battery was based on the sensitivity of the measurements to nonselective GABAergic treatments, and on the coverage of a wide range of different CNS domains (Table 2). This approach enabled us to identify unique effect profiles for pharmacologically distinct GABAergic treatments, including (1) traditional, pharmacologically nonselective, full GABAergic compounds at their clinical dose(s) (i.e., lorazepam 2 mg and alprazolam 1 mg), (2) a marketed GABAergic compound with high *α*_1_-subtype affinity (i.e., zolpidem 10 mg), and (3) several novel, *α*_2,3_-subtype selective GABAergic compounds at different investigational doses.

The new class of partial subtype selective GABA agonists was expected to be anxiolytic but less sedating and cognition impairing, as indicated by the preclinical* in vitro* and* in vivo* data. The anxiolytic effects of nonselective GABAergic agonists are accompanied by somnolence, impaired locomotion, and cognitive disturbance. These clinical side effects are reflected by the pharmacodynamics effects of lorazepam or alprazolam on VAS_alertness_ (measure of subjective sedation), body sway (measure of postural instability), and adaptive tracking (measure of visuomotor coordination). Memory testing was not performed frequently and consistently enough to allow a comparative analysis among the different compounds. However, the original publication of the TPA023-study provides indications that the partial subtype selective GABA agonist has fewer cognitive effects than the partial subtype selective GABA agonist. In this study, lorazepam 2 mg showed clear memory reductions, which did not occur with a dose of TPA023 1.5 mg that caused comparable SPV reductions [12].

Saccadic peak velocity (SPV) has previously been shown to be closely related to the anxiolytic doses of benzodiazepines [11], and SPV was therefore used as a reference parameter. As expected, SPV showed significant responses to almost every GABAergic compound investigated in these six studies [12–14]. In contrast to lorazepam or alprazolam, which influenced each output parameter of the saccadic eye movement test (i.e., SPV, saccadic reaction time, and inaccuracy), the *α*_1_-(zolpidem) or *α*_2,3_-subtype selective GABAergic compounds (TPA023, TPACMP2, SL65.1498) only affected SPV.

At their highest investigational dose, the effect size of TPA023 and TPACMP2 on SPV was comparable to the effects observed with lorazepam or alprazolam, whereas the effect of SL65.1498 was only marginally significant on SPV. In almost all these cases, the impact on other CNS effects was lower. This by itself is an indication of pharmacological selectivity, but a comparison based merely on overall or maximum effects could obscure some of the more subtle pharmacological differences (like the findings of SL65.1498 study) when the pharmacodynamic biomarker is less sensitive to the drug or if the dose of a drug is subtherapeutic. The relationships between the ΔSPV-effects and other pharmacodynamic (ΔPD) effects provide a complete profile of the differential effects, at each time point after drug administration. These outputs reflect the degree of *α*_2,3_ selectivity and may therefore also be indicators for anxioselectivity. Based on these perceptions, a GABAergic compound with “flat” regression lines in the ΔSPV-relative plotting graphs would show anxiolysis with reduced off-target effects in clinical settings. For most of the novel compounds described in this overview, there are no clinical reports of anxiolytic effects or improved tolerability. However, a recent article on TPA023, the oldest compound in this meta-analysis, reported reduced anxiety in a preliminary clinical trial at doses that were also used in our pharmacodynamic studies [4]. No detailed comparative information is available on the therapeutic window in these clinical trials.

We found that the ΔSPV-relative effect profiles of *α*_2,3_ subtype-specific GABAergic compounds are similar among each other but different from lorazepam 2 mg. The absolute slopes of the regression lines for the ΔSPV-ΔPD relations are generally lower with the selective GABA_A_ agonists than with the benzodiazepines. The results of alprazolam were comparable to lorazepam, which provides additional confidence that the analyses reflect pharmacological differences as well as similarities. Zolpidem seemed to be the only major exception, since this *α*_1_ subtype-selective GABAergic compound produced considerably steeper average slopes for certain ΔSPV-relative profiles than lorazepam or alprazolam, whereas the statistical population model did not reveal statistically significant differences between zolpidem and the benzodiazepines. This could reflect a limitation of the population model for ΔSPV-ΔPD relationships, which was chosen to be simple and unbiased, but necessarily had to ignore some rather complex individual response relationships. The analyses were based on linear slope estimates without a fixed intercept. In reality, however, all individual data points started at a fixed intercept (at *T* = 0, when ΔSPV and ΔPD were both zero), and, in many cases, the ΔSPV-ΔPD relationships were not linear, and zolpidem even formed loops when the SPV effect displayed a different time course than the PD effect. In almost all other cases, however, the statistical analyses and the graphical representations of the average relationships provide accurate representations of the individual plots.

This meta-analysis indicates that comparisons of ΔSPV-ΔPD profiles are able to identify pharmacological differences between subtype selective and nonselective GABA_A_ agonists. A comparison of ΔSPV-ΔPD profiles with the underlying pharmacological properties was refuted by the very small number of compounds for which this could be compared. Nonetheless, strong relationships (with an *R*-value of 0.93) between the *α*_1_/*α*_2_-ratios of the four compounds for which this could be determined and their ΔSPV-ΔVAS_alertness_ ratios. Clearly this remains to be confirmed with larger numbers of compounds. Still, the consistent ΔSPV-relative profiles of the selective GABAergic compounds suggest potential links between the preclinical profiles and the ΔSPV-relative pharmacodynamics profiles of these compounds. Moreover, TPACMP2 showed a distinct ΔSPV-ΔVAS_alertness_ relation but shared a similar ΔSPV-ΔSway relation with the other *α*_2,3_-subtype-selective GABAergic agonists. The relatively large amount of sedation with TPACMP2 could reflect the relatively high ratio of *α*_1_/*α*_2_-efficacy of TPMCMP2 compared to the other compounds. Similarly, the large efficacy of zolpidem is compatible with its steep ΔSPV-ΔVAS_alertness_ ratio and the strong hypnosedative effect of this z-hypnotic in the clinic.

## 5. Conclusion

TPA023, TPACMP2, and SL65.1498 are members of the novel experimental drug family of *α*_2,3_-subtype selective receptor agonists. *In vitro* pharmacological properties of these compounds indicate higher binding affinity and relative efficacy at the *α*_2,3_-subunits. *In vivo *preclinical studies with animal models translated such pharmacological properties into potential of anxiolysis and relatively reduced off-target effects in comparison with nonselective full GABAergic agonists like benzodiazepines.

The Neurocart battery is a collection of validated tests amenable to the effects of various CNS-acting drugs. Components of this battery were shown to be sensitive to different rapid-onset CNS effects of the benzodiazepines, in which reduction of saccadic peak velocity displays features of a GABAergic anxiolytic biomarker, whereas impairments of body sway, adaptive tracking, and memory are translated to effects that are less desirable for an anxiolytic drug. Most novel GABAergic compounds showed dose-dependent responses to saccadic peak velocity but did not affect the other CNS effects to the same extent, indicative of the pharmacoselectivity of these new compounds. Moreover, the ΔSPV-relative effect profiles provide information about dose potency and effect specificity. This battery is suitable to not only present the general depressive effects of benzodiazepines but also demonstrate the pharmacological selectivity and specificity of the novel GABAergic compounds. Comparative effect profiling as used in these studies can provide clear indications for the pharmacological selectivity and specificity of novel GABAergic compounds in healthy volunteers. This is a valuable approach for the early drug development of this new drug class, which will hopefully contribute novel anxiolytics with an improved therapeutic window to patients with anxiety disorders.

## Figures and Tables

**Table 1:** *In vitro* pharmacological property of the GABAergic compounds.

Compound	*α*_1_		*α*_2_		*α*_3_		*α*_5_		*α*_1_/*α*_2_-ratio
	*K*_*i*_ (nM)	Efficacy° (%)	*K*_*i*_ (nM)	Efficacy°(%)	*K*_*i*_ (nM)	Efficacy°(%)	*K*_*i*_ (nM)	Efficacy° (%)	
TPA023* [26]	0.27	0^#^	0.31	11	0.19	21	0.41	5	0
TPACMP2* [13]	0.22	18	0.40	23	0.21	45	0.23	18	0.78
SL65.1498^#^ [28]	17	45	73	115	80	83	215	48	0.39
Zolpidem	20 [29]	75*^§^* [30]	400 [29]	78^§^ [30]	400 [29]	80^§^	5000 [29]	9^§^ [30]	0.96

°Relative efficacy is defined as the extent of the potentiation of GABA-A EC20-equivalent current produced by the compound compared to that produced by a nonselective full agonist (chlordiazepoxide/diazepam).

*Mean values of 3 experiments in Xenopus oocytes with human recombinant *αβ*3*γ*2 receptors; efficacy relative to chlordiazepoxide.

^#^Mean values of 3 experiments in hek293 cells with recombinant rat receptors *αβ*2*γ*2; efficacy relative to chlordiazepoxide.

^§^Mean values of 3 experiments in Xenopus oocytes with human recombinant *αβ*2*γ*2 receptor; efficacy relative to diazepam.

**Table 2:** Component tests of the Neurocart battery and the related CNS domains.

Neurocart test	Targeted function	Related CNS areas
Saccadic eye movement	Neurophysiologic function	Superior colliculus, substantia nigra, amygdala
Smooth pursuit	Neurophysiologic function	Midbrain
Adaptive tracking	Visuomotor coordination	Neocortex, basal nuclei, brain stem, cerebellum
Body sway	Balance	Cerebellum, brain stem
Visual verbal learning test (VVLT)	Memory	Hippocampus
VAS Bond and Lader	Alertness, mood, calmness	Cortex, prefrontal cortex
VAS Bowdle	Feeling high, internal and external perception	Cortex, prefrontal cortex, amygdala

**Table 3:** Use of pharmacodynamic tests in each study.

Study	CHDR99112	CHDR0102	CHDR0105	CHDR0614	CHDR0407
compound	TPA023	TPACMP2	SL65.1498	Alprazolam	Zolpidem
comparator	Lorazepam	Lorazepam	Lorazepam	NA	NA
SEM	Done	Done	Done	Done	Done
Sway	Done	Done	Done	Done	Done
VAS BL	Done	Done	Done	Done	Done
Smooth	ND	ND	Done	Done	Done
Track	ND	ND	ND	Done	Done

ND: not done; NA: not applicable; SEM: saccadic eye movement; Smooth: smooth pursuit; Sway: body sway; VAS BL: VAS Bond and Lader; Track: adaptive tracking.

**Table 4:** Results of the linear model for saccadic peak velocity change from baseline and log body sway change from baseline by treatment with treatment by SPV change from baseline as interaction.

Treatment	ΔSPV-relative relation	Item	Estimate of treatment	Estimate of lorazepam	*P* value
TPA023 1.5 mg	ΔSway-ΔSPV	Slope	−0.00048	−0.00305	<0.0001
		Intercept	−0.01316	0.1292	<0.0001
	ΔVAS_alertness_-ΔSPV	Slope	0.03312	0.126	0.0001
		Intercept	0.4551	−4.4739	0.0021

TPACMP2 0.75 mg	ΔSway-ΔSPV	Slope	−0.00027	−0.00305	<0.0001
		Intercept	0.03784	0.1292	0.0009
	ΔVAS_alertness_-ΔSPV	Slope	0.09884	0.126	0.2525
		Intercept	−1.4465	−4.4739	0.0397

SL65.1498 25 mg	ΔSway-ΔSPV	Slope	−0.00128	−0.00305	0.0003
		Intercept	0.0222	0.1292	<0.0001
	ΔVAS_alertness_-ΔSPV	Slope	0.04193	0.126	0.0009
		Intercept	0.2453	−4.4739	<0.0001
	ΔSmooth-ΔSPV	Slope	0.01554	0.1099	<0.0001
		Intercept	−1.4483	−6.2553	<0.0001

Alprazolam 1 mg	ΔSway-ΔSPV	Slope	−0.00204	−0.00305	0.0667
		Intercept	0.001788	0.1292	<0.0001
	ΔVAS_alertness_-ΔSPV	Slope	0.0734	0.126	0.0763
		Intercept	−0.628	−4.4739	0.0254
	ΔTrack-ΔSPV	Slope	0.0747	0.0572	0.1545
		Intercept	0.3023	−4.0742	<0.0001
	ΔSmooth-ΔSPV	Slope	0.08077	0.1099	0.2808
		Intercept	−1.4025	−6.2553	0.0002

Zolpidem 10 mg	ΔSway-ΔSPV	Slope	−0.0033	−0.00305	0.7336
		Intercept	0.06014	0.1292	0.0127
	ΔVAS_alertness_-ΔSPV	Slope	0.1526	0.126	0.5231
		Intercept	−3.2697	−4.4739	0.5219
	ΔTrack-ΔSPV	Slope	0.0489	0.0572	0.6240
		Intercept	−0.9123	−4.0742	<0.0001
	ΔSmooth-ΔSPV	Slope	0.09771	0.1099	0.7412
		Intercept	−3.8439	−6.2553	0.0815

## References

[1] Seligman MEP, Walker EF, Rosenhan DL (2000). *Abnormal Psychology*.

[2] Eison AS, Mullins UL (1995). Regulation of central 5-HT_2A_ receptors: a review of in vivo studies. *Behavioural Brain Research*.

[3] Wong G, Skolnick P (1992). High affinity ligands for “diazepam-insensitive” benzodiazepines receptors. *European Journal of Pharmacology—Molecular Pharmacology Section*.

[4] Atack JR (2009). Subtype-selective GABA_A_ receptor modulation yields a novel pharmacological profile: the design and development of TPA023. *Advances in Pharmacology*.

[5] McKernan RM, Rosahl TW, Reynolds DS (2000). Sedative but not anxiolytic properties of benzodiazepines are mediated by the GABA_A_ receptor *α*1 subtype. *Nature Neuroscience*.

[6] Rowlett JK, Platt DM, Lelas S, Atack JR, Dawson GR (2005). Different GABA_A_ receptor subtypes mediate the anxiolytic, abuse-related, and motor effects of benzodiazepine-like drugs in primates. *Proceedings of the National Academy of Sciences of the United States of America*.

[7] Knabl J, Witschi R, Hösl K (2008). Reversal of pathological pain through specific spinal GABA_A_ receptor subtypes. *Nature*.

[8] Knabl J, Zeilhofer UB, Crestani F, Rudolph U, Zeilhofer HU (2009). Genuine antihyperalgesia by systemic diazepam revealed by experiments in GABA_A_ receptor point-mutated mice. *Pain*.

[9] Atack JR, Bayley PJ, Seabrook GR, Wafford KA, McKernan RM, Dawson GR (2006). L-655,708 enhances cognition in rats but is not proconvulsant at a dose selective for *α*
_5_-containing GABA_A_ receptors. *Neuropharmacology*.

[10] Ballard TM, Knoflach F, Prinssen E (2009). RO4938581, a novel cognitive enhancer acting at GABAA *α*
_5_ subunit-containing receptors. *Psychopharmacology*.

[11] De Visser SJ, Van Der Post JP, De Waal PP, Cornet F, Cohen AF, Van Gerven JMA (2003). Biomarkers for the effects of benzodiazepines in healthy volunteers. *British Journal of Clinical Pharmacology*.

[12] De Haas SL, De Visser SJ, Van Der Post JP (2007). Pharmacodynamic and pharmacokinetic effects of TPA023, a GABA_A_
*α*
_2,3_ subtype-selective agonist, compared to lorazepam and placebo in healthy volunteers. *Journal of Psychopharmacology*.

[13] De Haas SL, De Visser SJ, Van Der Post JP (2008). Pharmacodynamic and pharmacokinetic effects of MK-0343, a GABA_A_
*α*
_2,3_ subtype selective agonist, compared to lorazepam and placebo in healthy male volunteers. *Journal of Psychopharmacology*.

[14] De Haas SL, Franson KL, Schmitt JAJ (2009). The pharmacokinetic and pharmacodynamic effects of SL65.1498, a GABA_A_ 2,3 selective agonist, in comparison with lorazepam in healthy volunteers. *Journal of Psychopharmacology*.

[15] De Haas SL, Schoemaker RC, Van Gerven JMA, Hoever P, Cohen AF, Dingemanse J (2010). Pharmacokinetics, pharmacodynamics and the pharmacokinetic/ pharmacodynamic relationship of zolpidem in healthy subjects. *Journal of Psychopharmacology*.

[16] Van Steveninck AL, Van Berckel BNM, Schoemaker RC, Breimer DD, Van Gerven JMA, Cohen AF (1999). The sensitivity of pharmacodynamic tests for the central nervous system effects of drugs on the effects of sleep deprivation. *Journal of Psychopharmacology*.

[17] Baas JMP, Mol N, Kenemans JL (2009). Validating a human model for anxiety using startle potentiated by cue and context: the effects of alprazolam, pregabalin, and diphenhydramine. *Psychopharmacology*.

[18] Van Steveninck AL, Verver S, Schoemaker HC (1992). Effects of temazepam on saccadic eye movements: concentration-effect relationships in individual volunteers. *Clinical Pharmacology and Therapeutics*.

[19] Van Steveninck AL, Schoemaker HC, Pieters MSM, Kroon R, Breimer DD, Cohen AF (1991). A comparison of the sensitivities of adaptive tracking, eye movement analysis, and visual analog lines to the effects of incremental doses of temazepam in healthy volunteers. *Clinical Pharmacology and Therapeutics*.

[20] Bittencourt PRM, Wade P, Smith AT, Richens A (1981). The relationship between peak velocity of saccadic eye movements and serum benzodiazepine concentration. *British Journal of Clinical Pharmacology*.

[21] Baloh RW, Sills AW, Kumley WE, Honrubia V (1975). Quantitative measurement of saccade amplitude duration, and velocity. *Neurology*.

[22] Norris H (1971). The action of sedatives on brain stem oculomotor systems in man. *Neuropharmacology*.

[23] Bond A, Lader M (1974). The use of analogue scales in rating subjective feelings. *British Journal of Medical Psychology*.

[24] Van Steveninck AL, Gieschke R, Schoemaker HC (1993). Pharmacodynamic interactions of diazepam and intravenous alcohol at pseudo steady state. *Psychopharmacology*.

[25] Van Steveninck AL, Wallnöfer AE, Schoemaker RC (1997). A study of the effects of long-term use on individual sensitivity to temazepam and lorazepam in a clinical population. *British Journal of Clinical Pharmacology*.

[26] Atack JR, Wafford KA, Tye SJ (2006). TPA023 [7-(1,1-dimethylethyl)-6-(2-ethyl-2H-1,2,4-triazol-3-ylmethoxy)-3- (2-fluorophenyl)-1,2,4-triazolo[4,3-b]pyridazine], an agonist selective for *α*2- and 3-containing GABA_A_ receptors, is a nonsedating anxiolytic in rodents and primates. *Journal of Pharmacology and Experimental Therapeutics*.

[27] Borland RG, Nicholson AN (1984). Visual motor co-ordination and dynamic visual acuity. *British Journal of Clinical Pharmacology*.

[28] Griebel G, Perrault G, Simiand J (2001). SL651498: an anxioselective compound with functional selectivity for *α*
_2_- and *α*
_3_-containing *γ*-aminobutyric acid_A_
(GABA_A_) receptors. *Journal of Pharmacology and Experimental Therapeutics*.

[29] Crestani F, Martin JR, Möhler H, Rudolph U (2000). Mechanism of action of the hypnotic zolpidem in vivo. *British Journal of Pharmacology*.

[30] Sanna E, Busonero F, Talani G (2002). Comparison of the effects of zaleplon, zolpidem, and triazolam at various GABA_A_ receptor subtypes. *European Journal of Pharmacology*.

